# Barraquer Simons Syndrome: Case Series and Review of Surgical Treatments for Facial Lipodystrophy

**DOI:** 10.1007/s00266-025-04794-z

**Published:** 2025-03-24

**Authors:** Meryem Hamam, Atilla Adnan Eyuboglu, Mustafa Tonguc Isken

**Affiliations:** 1 Hamidiye International School of Medicine, University of Health Sciences, Istanbul, Turkey; 2 Department of Plastic and Reconstructive Surgery, Faculty of Medicine, Bahcelievler Memorial Hospital, Arel University, Istanbul, Turkey; 3 Department of Plastic and Reconstructive Surgery, Faculty of Medicine, Bahcelievler Memorial Hospital, Bahcesehir University, Istanbul, Turkey

**Keywords:** Barraquer Simons syndrome, Facial lipoatrophy, Lipodystrophy

## Abstract

**Background:**

Barraquer Simons Syndrome (BSS) is a subtype of idiopathic acquired lipodystrophy, wherein patients—mostly female—lose subcutaneous fat in the upper half of the body starting in childhood or puberty. A disproportionate fat allocation can be seen in the trochanteric region and thighs.

**Methods:**

Along with three cases of lipoatrophy treated at our hospital, we conducted a comprehensive review and presented the treatments in the literature for improving bilateral facial lipodystrophy.

**Results:**

Flaps and autologous fat grafting procedures were favored across the literature, and regardless of the intervention of choice, patients reported satisfaction and improved life prospects.

**Conclusion:**

The loss of facial fat is especially afflicting for patients as the bilateral lipoatrophy imparts an aged, cachexic look which negatively affects not only their self-image but their social status as well. Alternatives to approach lipoatrophy vary in their invasiveness, permanency, and feasibility; thus, treatments must be thoroughly tailored to each patient’s needs and expectations.

- Barraquer Simons Syndrome is a rare subtype of lipodystrophy primarily affecting females, leading to fat loss in mainly the upper body.
- The negative social impact of the syndrome necessitates intervention, and patients receiving appropriate treatment reported improved psychosocial parameters.
- Treatment plans should consider each patient's specific needs and expectations, balancing invasiveness, permanency, and feasibility of the procedures.

**Level of Evidence IV:**

This journal requires that authors assign a level of evidence to each article. For a full description of these Evidence-Based Medicine ratings, please refer to the Table of Contents or the online Instructions to Authors  www.springer.com/00266.

## Introduction

Partial Lipodystrophy is a subtype of lipodystrophic diseases that present with regional subcutaneous adipose tissue atrophy [1]. They can be either congenital as in Familial Partial Lipodystrophy or acquired as in Barraquer–Simons Syndrome (BSS) [2]. The latter, first described by Barraquer–Roviralta more than a century ago, is the focus of this current review [1, 3]. Acquired Partial Lipodystrophy (APL) is frequently associated with autoimmune diseases such as systemic lupus erythematosus and dermatomyositis, though the exact mechanisms underlying pathogenesis are yet to be identified [4]. APL can be of unknown etiology as in BSS, induced by Highly Active Anti-Retroviral Therapy (HAART) or associated with total body irradiation and hematopoietic stem cell transplant [2, 5]. It is four times more common in women, and the onset of the disease is frequently around ages 5–15 years [3]. The loss of adipose tissue occurs slowly in a symmetrical manner over months, and it is more pronounced in the upper body—specifically the face, trunk, and arms [1, 3]. The described craniocaudal pattern of atrophy is accompanied by increased fat deposits in the lower body [1, 3]. Typically, patients express concern about weight gain non-responsive, emaciated appearance on the face, which is ultimately associated with a prematurely aged look [3].

Barraquer–Simons Syndrome patients may share common external features such as higher than average height, increased size of genitalia, cutaneous pigmentation, and virilization [3]. Internally, the patients may present with hepatomegaly, diabetes, and hyperlipidemia, though metabolic manifestations are less prevalent in BSS compared with other lipodystrophies [4, 5].

Of particular importance, more than half of the patients have low serum C3 caused by C3 nephritic factor, which is associated with the nephropathy commonly encountered in these patients [2]. In fact, around 25% of BSS-afflicted patients develop nephropathies such as membranoproliferative glomerulonephritis type II (MPGN), with some progressing to end-stage renal disease and requiring renal transplant [2].

If BSS is suspected in a patient, the presence of autoimmune diseases, as well as low serum C3 and detectable C3 nephritic factor helps in confirming the diagnosis alongside the regionality of lipoatrophy [1]. BSS can also follow microbial infections in childhood such as measles, highlighting the vitality of obtaining the clinical history of patients in depth [4]. Barraquer–Simons Syndrome is not to be confused with Parry-Romberg Syndrome (PRS), which also presents with facial adipose atrophy within the first two decades of life. Unlike in BSS, patients with PRS experience unilateral atrophy of not only the subcutaneous fat, but also of the skin, musculature, and, in severe cases, bone [6]. Barraquer–Simons Syndrome should also be differentiated from anorexia nervosa, as facial lipoatrophy within the first 15 years of life imparts a starving appearance [5]. Notably, BSS may go undiagnosed as some patients may mistakenly attribute the external features of the disease to their “normal body type”. Thus, the role the physician plays in recognizing the disease is further underlined, considering the associated comorbidities that require regular management.

Integrative physical and mental treatment is crucial, as patients, besides the metabolic complications and nephropathy, experience emotional distress about their appearance, complaining that the disease’s outward manifestations affect their social life and employment [7, 8].

With no definitive treatment for BSS, approaches to improving life quality include diet modifications and regular exercise regimes. Treatments tailored for metabolic diseases and, of specific relevance to BSS, nephropathy are of importance [2, 9]. As we will demonstrate in the current review, the role the plastic surgeon plays is important as surgical treatments tend to improve patient well-being and self-esteem. Hesitancy in opting for surgical treatment is associated with concerns regarding the long-term outcomes of the procedures, especially considering that treatments are done early in life and patients worry about possible insufficiency. Hence, with this review, we aim to weigh the effectiveness of surgical treatments in BSS through a systematic review, analysis of the literature, and our experience with patients presenting to our clinic.

## Patients and Methods

Three patients presented to our hospital with BSS between January 2019 and December 2023. Patients had the classical presentation of the disease, severe lipoatrophy of the upper half of the body and especially of the face.

All patients were gone under meticulous evaluation including physical, radiological, and laboratory examinations. Written informed consent forms are obtained from the patients. Ethical approval has been obtained from the local ethical committee. Patients are presented to demonstrate our clinical approach and possible solutions for BSS.

## Patients

### Patient 1

**Fig. 1:**
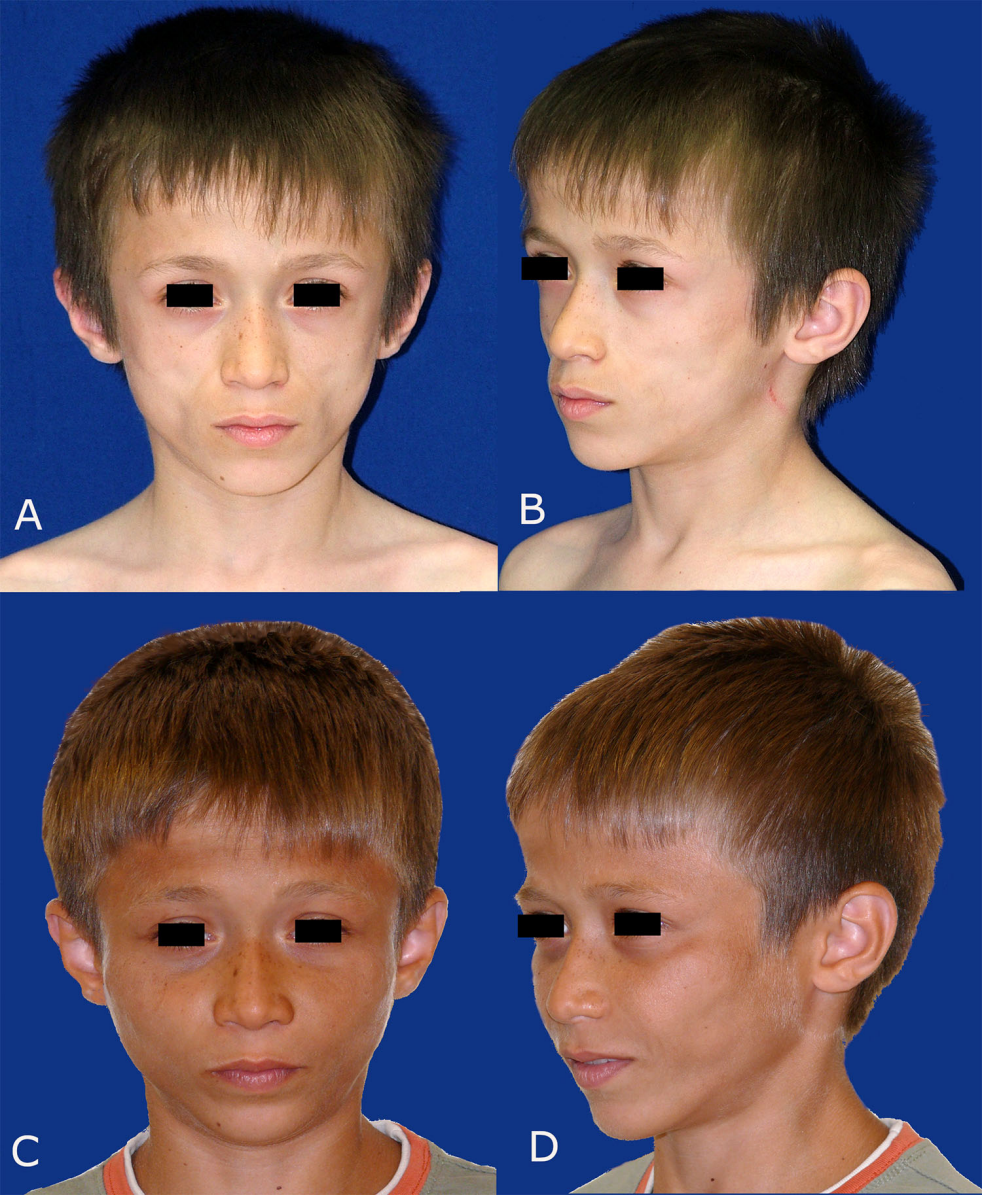
A 7 years old patient with facial lipodystrophy treated using medpor implants to fix the facial contour defects. **A** Preoperative front view, **B** Preoperative oblique view, **C** Postoperative front view, **D** Postoperative oblique view

A 7-year-old boy was referred to our clinic due to concerns about his appearance, which had led to ridicule from his peers. His facial features initially suggested a case of malnourishment. However, after thorough examinations and laboratory analyses, the patient was found to be in overall good health (Fig. 1A, B). While fat injections were initially considered as a treatment option, it was determined that the bony structure in the zygomatic region would be better addressed with implants.

**Fig. 2:**
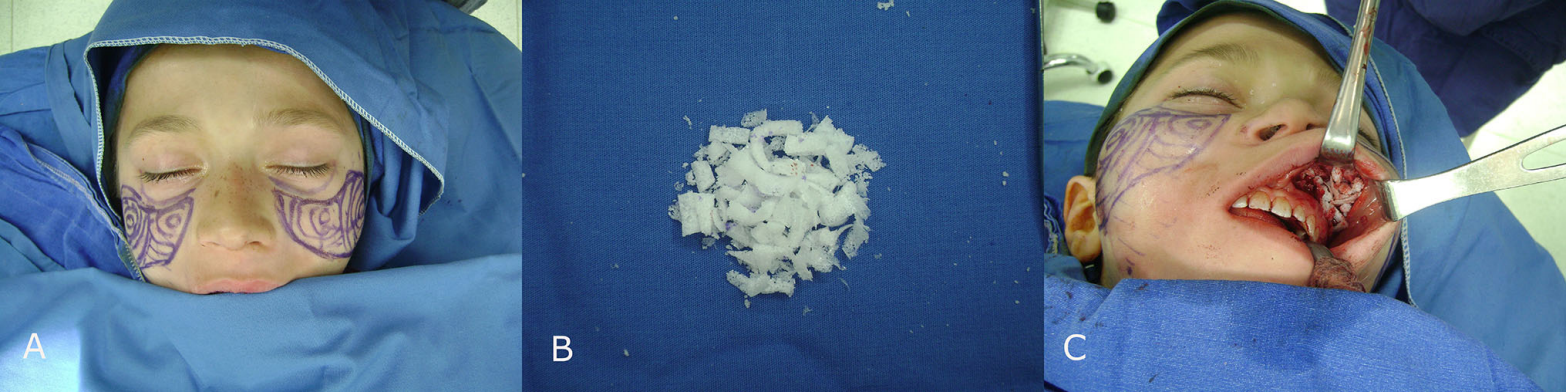
Intraoperative image of a patient treated using medpor implant pieces. **A** Intraoperative image showing the marking done preoperatively, **B** Image of minced Medpor® implants, **C** Image of the patient after implantation of medpor implants thru intraoral approach

We chose Medpor® implants to correct the facial contour defects. Preoperative markings were made to outline the precise contours of the zygoma and arcus zygomaticus (Fig. 2A). To minimize the risk of infection, we adhered to the standard prophylactic antibiotic regimen and irrigated the implants with an antibiotic solution. The procedure was planned using an intraoral approach with gingivobuccal incisions. Tumescent infiltration was applied for hydro dissection, followed by the elevation of the periosteum to create a pocket for the implant. To improve vascularization and adaptation, the Medpor® implant was minced (Fig. 2B) and used to fill the prepared pocket (Fig. 2C). The incision lines were then closed with watertight, monofilament absorbable sutures. The operation was completed without complications, and the patient healed without any issues related to wound healing or infection. During the one-year follow-up period, no complications or adverse events were observed. The final outcome was satisfactory. (Fig. 1C, D).

### Patient 2

**Fig. 3:**
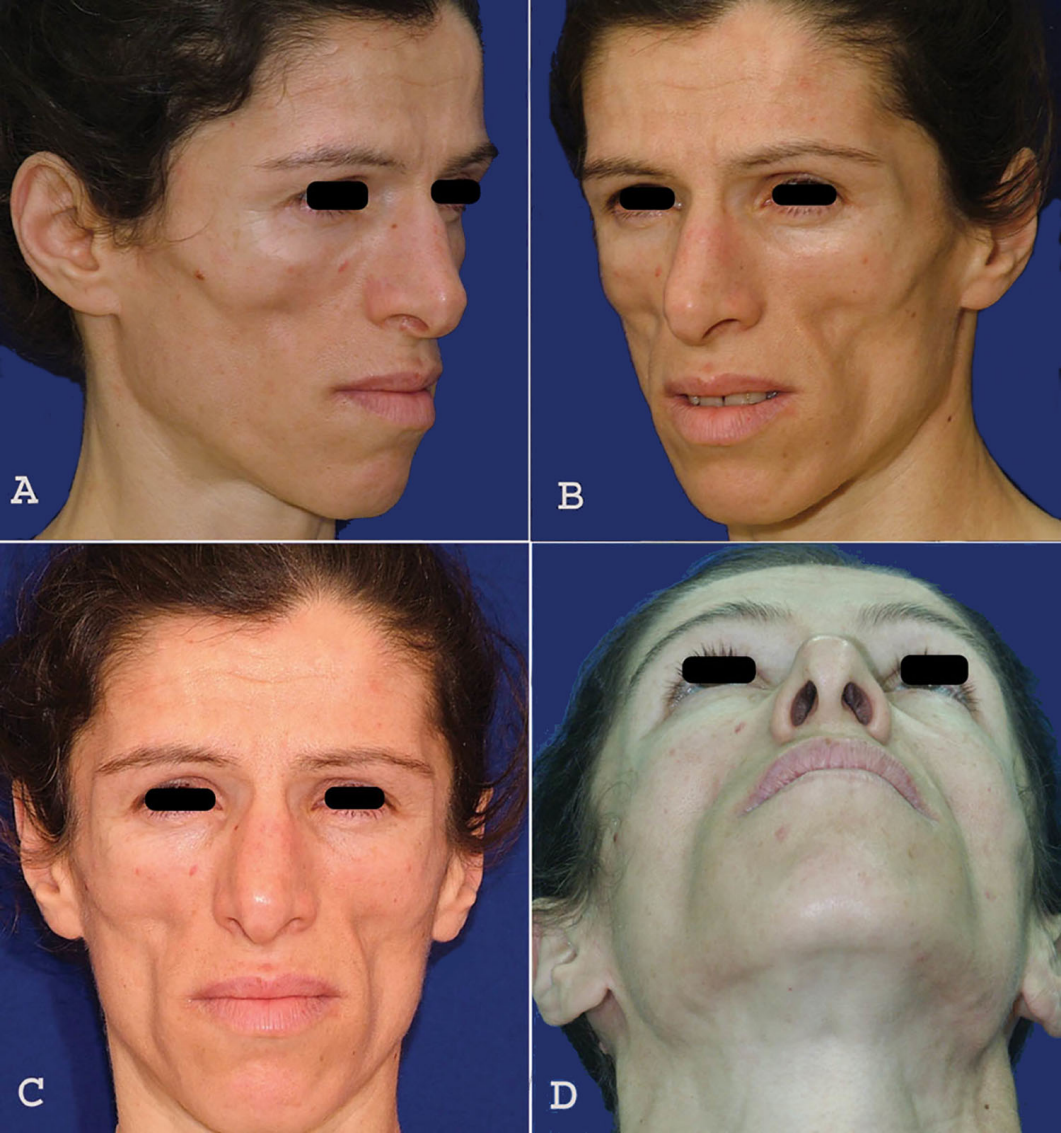
Preoperative images of a 34 years old female with severe facial lipoatrophy. **A** Preoperative right oblique view, **B** Preoperative left oblique view, **C** Preoperative front view, **D** Preoperative right worms eye view

The second patient, a female aged 34 years old had severe facial lipoatrophy which was a cause of distress, as her appearance was often mistaken for anorexia nervosa—or, in some instances acquired immunodeficiency syndrome (AIDS) (Fig. 3). This faulty attribution of the underlying causes of her appearance ultimately led to social problems.

**Fig. 4:**
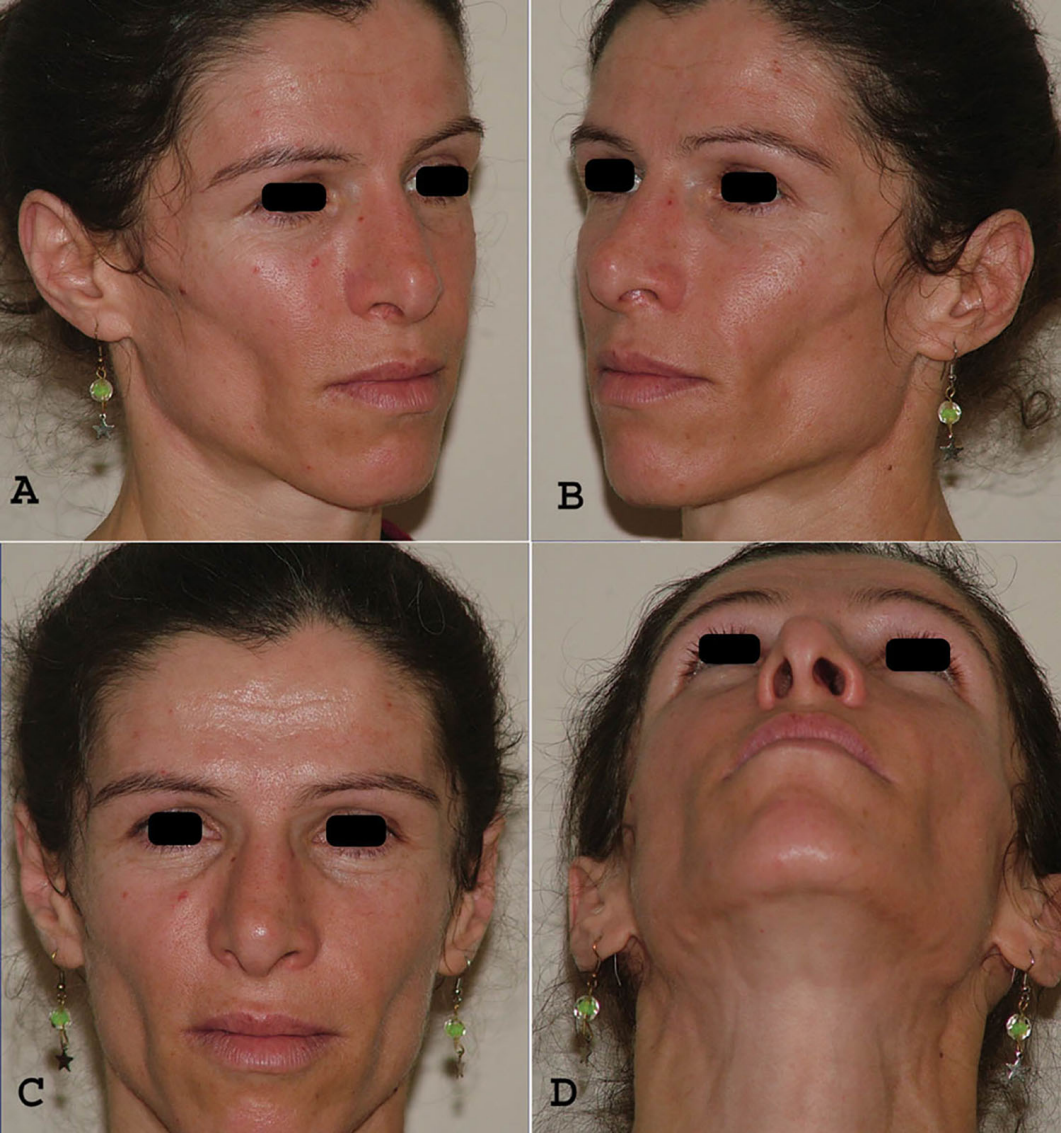
Postoperative images of a 34 years old female with severe facial lipoatrophy. **A** Postoperative right oblique view, **B** Postoperative left oblique view, **C** Postoperative front view, **D** Postoperative right worms eye view

For this patient, we selected spongiose bone grafts to address the facial volume loss. The procedure was planned using an intraoral approach with gingivobuccal incisions. Tumescent infiltration was applied for Hydro dissection, followed by the elevation of the periosteum to create a pocket for the bone grafts. The spongiose bone grafts were harvested from the iliac crest using an ultrasonic cutter (Piezo®) to remove the cortical segment, and the grafts were then adapted to their anatomical location. The donor site was closed anatomically, and the bone grafts were used to fill the prepared pocket. The intraoral incisions were closed with watertight, monofilament absorbable sutures. The operation was completed without complications, and the patient healed well, with no issues related to wound healing or infection. Although the spongy bone grafts exhibited some resorption, this is a common and expected occurrence with any type of graft. Despite this, we achieved satisfactory results (Fig. 4).

## Patient 3

**Fig. 5:**
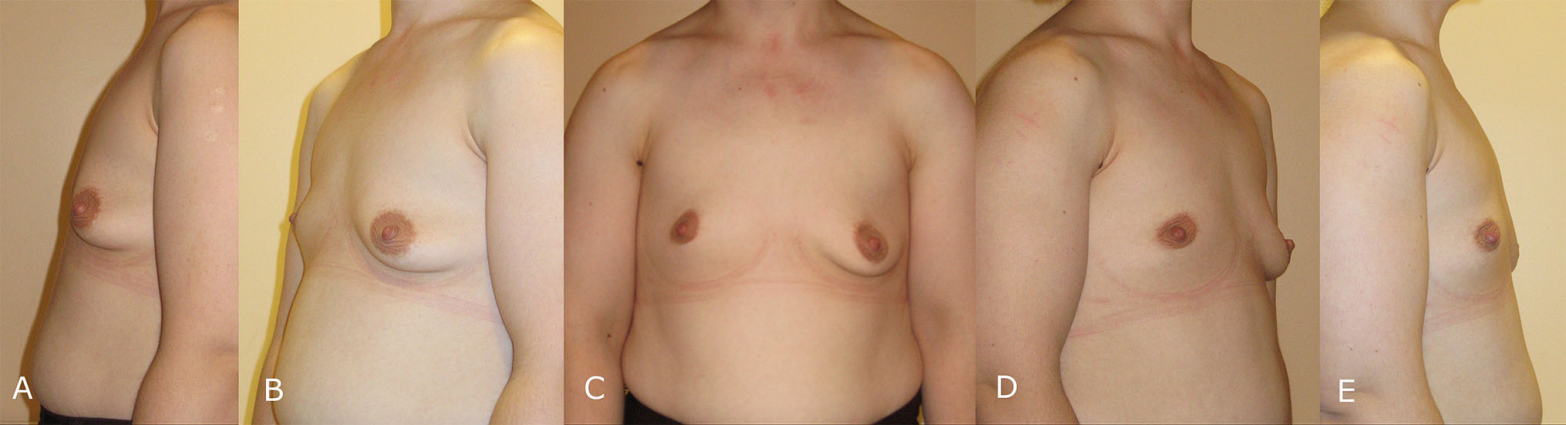
Preoperative images of a 29 years old female with facial lipoatrophy combined with breast atrophy. **A** Preoperative left side view, **B** Preoperative left oblique view, **C** Preoperative front view, **D** Preoperative right side view, **E** Preoperative right side view

A female aged 29, complained of atrophic breast tissue. For her facial lipoatrophy, she underwent lipofilling at another clinic. Thorough examinations and laboratory analyses, the patient was found to be in overall good health. She had asymmetries on the breast forming breast ptosis, especially on the left side (Fig. 5).

**Fig. 6:**
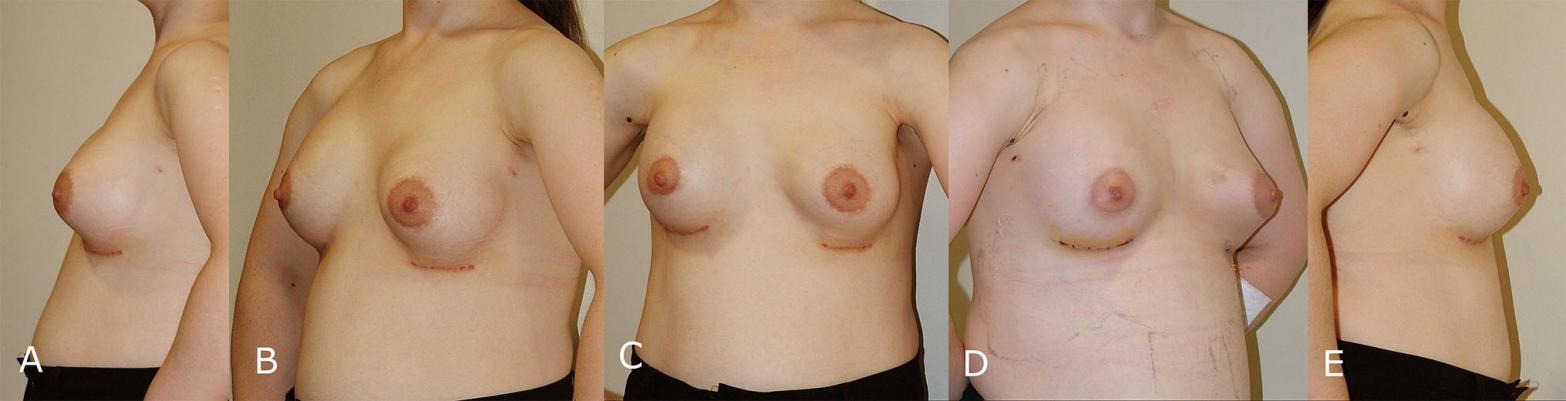
Postoperative images of a 29 years old female with facial lipoatrophy combined with breast atrophy. **A**: Postoperative left side view, **B** Postoperative left oblique view, **C** Postoperative front view, **D** Postoperative right side view, **E** Postoperative right side view

We decided to use silicone implants to address the atrophy. Preoperative markings were made, and the implants were planned to be inserted through an inframammary fold incision. A pocket was created in the subfascial plane of the pectoralis major muscle. Sizers were used to determine the appropriate implant size. The deformity was corrected using 275 cc Mentor® moderate plus profile silicone implants. Hemostasis was achieved, and the incisions were closed in anatomical layers. The patient wore a supportive bra for three weeks post-procedure. We achieved satisfactory results (Fig. 6).

## Methods: Literature Review

We searched PubMed, Scopus, and Cochrane databases for retrieval of studies regarding surgical treatment methods for acquired partial lipodystrophy. Following the Preferred Reporting Items for Systematic Reviews and Meta-Analysis (PRISMA) 2020 standards, we conducted a comprehensive view of the literature and screened for eligible studies to include in our review [10].

The search key used for title and abstract screening in the databases was ((((lipodystrophy[Title/Abstract]) AND(surgical[Title/Abstract])) OR ((lipodystrophy[Title/Abstract]) AND ((surgery[Title/Abstract]) OR (surgical[Title/Abstract]))))) OR (((Barraquer–Simons Syndrome[Title/Abstract]) AND ((surgery[Title/Abstract]) OR (surgical[Title/Abstract])))) OR ((Barraquer–Simons syndrome[Title/Abstract]) AND (surgical[Title/Abstract]))

Studies were included if they met the following criteria:

(i) Original research, case series or reports.
(ii) Aimed to investigate or present a surgical treatment methodology for acquired partial lipodystrophy.
(iii) And published in English.

Studies were excluded if

(i) They are reviews, letters to the editor,
(ii) They do not explore surgical treatment of acquired partial lipodystrophy directly (such as investigating diagnostic criteria and methods only)
(iii) The patients’ underlying cause for APL is HIV treatment.

The first Author extracted the data from the included studies and entered the collected information into a pre-piloted Excel spreadsheet. To ensure accuracy, other authors reviewed the data sheet, resolved any conflicting points, and validated the alignment of the data with the aim of our study. Extracted data was analyzed in accordance with the literature.

## Results

The authors employed further manual search through the web to ensure a broad scope. The search revealed 449 potentially relevant articles. The removal of duplicates yielded 243 studies, which were title-screened by two authors, using Rayyan Ai as the platform to agree on included articles.

Studies deemed eligible for abstract screening were screened by the same two authors. Following this, 63 studies remained eligible and were retrieved for full text screening, which was done according to pre-meditated inclusion/exclusion criteria, as shown in Table 1.

**Table 1:**
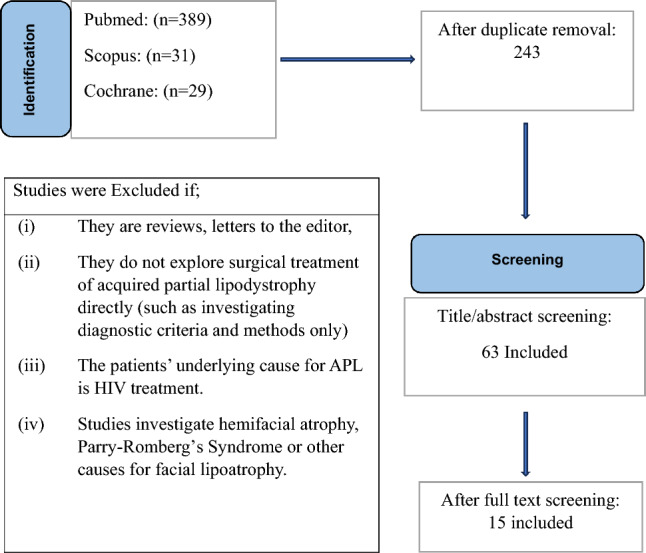
Search flow of the literature and criteria for exclusion/inclusion of the studies

After the full text screening, studies to be included were agreed on with the third author. In total, 16 papers fulfilled our inclusion criteria in this scoping review.

Baseline characteristics of the studies included author, year of publication, country, sample size, gender, mean age at presentation to the clinic, onset of disease, follow-up duration, previous treatments, treatment methodology in the study and patient outcomes along with complications (Table 2).

**Table 2:** Baseline characteristics of the studies included author, year of publication, country, sample size, gender, mean age at presentation to the clinic, onset of disease, follow-up duration, previous treatments, treatment methodology in the study and patient outcomes along with complications

Author	Year	Study design	Country	Sample size	Age at onset of lipodystrophy	Age at presentation (mean)	Follow-up duration	Previous treatment(s)	Treatment	Follow-up procedures or treatments	Outcome	Complications
Ower et al. [7]	1962	Case report	Canada	1 (F)	N/A	21	3 years	Hormone therapy	Free dermal fat auto- graft from the abdomen to the cheeks.	None	Patient was satisfied	None mentioned
Heidemann et al. [8]	2016	Case Report	Denmark	1 (F)	8	34	3 months after each treatment. 1st treatment—April 2014 2nd treatment—September 2014 3rd treatment—January 2016	None mentioned	Autologous fat grafting harvested from the lower extremities and injected into the face and breast	Yes, repeated treatments	Minimal loss of volume has been seen in the face or breasts at the follow-ups, and the patient expressed great satisfaction with treatment	None mentioned
Schmitz et al. [11]	2008	Retrospective	Luxembourg	7	14.6	The average delay after being diagnosed and the first-stage treatment was 16.9 years.	5.25 years.	None mentioned.	Free flap surgery (4) or lipofilling (3)	None mentioned in the study but recommended by the authors.	The overall results from free flap surgery were disappointing. cutaneous flaps showed better results than myocutaneous flaps. *for both BSS and PR. patients treated with classic lipofilling, an atrophy of 40% was present, yet this still gave better results than free flap surgery. This was because atrophy, following lipofilling, was uniform and ptosis did not develop.	one preoperative arterial thrombosis (no flap loss), hematomas, edema, section of the facial nerves, atrophy, and ptosis of the flap were encountered. *
Cortese et al. [12]	2000	Retrospective	Italy	4 (F)	N/A	N/A	Each patient had 1 treatment every 3 months for 3 to 4 times Follow-up was, every 6 months.	None mentioned.	Cheek and zygomatic bridge augmentation through autologous free fat transplantation (lipofilling)	None	Good results were obtained with fat resorption percentage ranging 75–85%	None mentioned.
Daurova et al. [13]	1985	Retrospective	Russia	22 (F)	6–17 (range)	18–50	2–5 years	None mentioned.	Bilateral subcutaneous injection of Elastosil MI. The site of injection was treated with collodium	None	Elastosil MI secures permanent cosmetic results. The compound is recommended for clinical use.	None mentioned.
Serra et al. [14]	1993	Case report	Spain	1 (F)	8	27	1 year	None mentioned.	Temporal muscle flap	None	The postoperative course was satisfactory and the facial defect was corrected with a high degree of symmetry.	None mentioned.
Hurwitz et al. [15]	1982	Case report	Israel	1 (F)	12	16	21 months following first operation	None mentioned.	Dermal fat graft	Yes, a following trimming operation	Patient was satisfied, mild asymmetry was present and fat resorption was within the standard limits	None mentioned.
Afra et al. [16]	2019	Case Report	India	1 (F)	12	27	6 months	None mentioned.	Hyaluronic acid filler	None	The volume deficit was corrected The patient had complete satisfaction and the outcome was maintained at her last follow‐up at sixth month.	None mentioned
Van der Wal et al. [17]	1998	Case report	Netherlands	1 (F)	5	35	1 year	None mentioned.	Temporal muscle flap	None	Stable and satisfactory.	None mentioned.
Endo et al. [18]	1994	Case report	Japan	1 (F)	30	38	20 months following the first surgery	None mentioned.	Radial forearm flap and free dermal fat grafts	6 months later the patient underwent the second stage operation (trimming) for the gravitational migration of the flap.	A natural appearance was achieved and the patient was satisfied. A mild asymmetry was still noticeable	None mentioned.
Coessens et al. [19]	1995	Case report	Belguim	1 (F)	After Puberty	38	15 months	Three fat transplants from legs to cheeks during the past 2 years without durable improvement of appearance.	Transverse rectus abdominis myocutaneous (TRAM) flap	None	Stable results were achieved.	None mentioned.
Goossens et al. [20]	2002	Case report	Belguim	1 (F)	Teenage years	28	1 year	None mentioned.	Transverse rectus abdominis myocutaneous (TRAM) flap	None	Eleven years following reconstruction, the result is satisfactory and the muscle volume is unchanged. The patient's appearance remains much im- proved, but we note a discrete temporal sagging, considered to be a normal evolution for the age of the	None mentioned.
Guelinckx et al. [21]	1999	Case series	Belguim	2 (F)	20 (1), 12 (2)	49	Patient 1 : not mentioned. patient 2 : 18 months	Patient 1: none mentioned patient 2: treated by fat cell injections into the cheek areas resulting in temporary improvement	Two free anterolateral thigh flaps.	None for patient 1 , for patient 2 Six months after the procedure, additional liposuction at the mandibular border was done to improve the cheek line.	Patient 1: The postoperative swelling resolved within several weeks, and soft cheeks were obtained. Patient 2: The post- operative course was uneventful, and after several weeks a smooth, soft facial appearance was obtained with low donor site morbidity.	For both patients Microscopic examination of the facial arteries and veins showed bilateralvasculitis and perivasculitis.
Okazaki et al. [22]	2012	Case Report	Japan	1 (F)	27	47	10 months	None.	1-stage bilateral restoration of the facial contour with 1 donor site and a single pair of vascular anastomoses (2 paddles of TAP adiposal flaps based on hemilateral thoracodorsal vessels).	Yes	Good blood supply was observed in both flaps after vascular anastomoses were completed. After a minor revision procedure including reduction of drooping adiposal tissue performed 6 months postoperatively, a good appearance was obtained.	None mentioned
Jeon et al. [24]	2020	Case Report	the UK	1 (F)	Teenage years	48	16 months after last procedure.	None mentioned.	Autologous fat transfer. Fat was harvested from both thighs. Fat was injected into the face and breast.	Yes, repeated treatments	Good fat retention in all areas, and the patient was satisfied with the results. At 16-month follow-up, the patient had experienced some fat resorption particularly affecting the cheeks and is subsequently on the waiting list for further fat transfer.	None mentioned
Davies et al. [25]	1989	Case series	USA	2 (F)	N/A	55	Patient 1: 1 year Patient 2: 3 years	Patient 1: None mentioned Patient 2: unsuccessful bilateral facial reconstruction with omental vascularized free flap at another institution	Patient 1: facial rhytidectomy, upper lip chemical peel, upper and lower blepharoplasties. Patient 2: facial rhytidectomy and dermis graft. septorhinoplasty and blepharoplasty.	None	Patient results were satisfactory.	None mentioned.

*F* Female, *N/A* Not Available, *TAP* Thoracodorsal Artery Perforator

*Results reported for both Barraquer Simons Syndrome and Parry-Romberg Syndrome patients

The included studies spanned a wide geography across Asia, Europe, and North America. The oldest included article was published in 1962 [7]. Most of the studies included were case reports; three studies were retrospectively designed [11–13]. As is the usual case with BSS, all patients were females with an onset of disease in childhood or puberty for the majority of the studies. Patients generally presented to the clinic in middle age, with the common complaint being facial lipoatrophy, which imparted a prematurely aged look [7]. Prior to treatment, all the studies emphasized the importance of the timing of the operations, hence, to avoid excess fat resorption post-treatment, the lipodystrophic process must have ended before the planned procedure [14]. Most of the treatments aimed to reconcile the emaciated facial appearance the disease causes, and in some patients, lipoatrophy of the breast tissue was treated as well [8].

The varying surgical treatment modalities preferred include autologous grafts, subcutaneous injections and fillers, rhytidectomy, autologous flaps and autologous free fat transplantation.

## Techniques

1. Autologous Grafts

In the two studies, where autologous grafts were utilized, the researchers opted for the abdomen and buttocks areas as donor sites [7, 15]. Both studies preferred free dermal-fat grafts. With free dermal-fat graft from the buttocks, following trimming procedure was required to remove the excess tissue. Both studies reported good patient satisfaction.

2. Fillers

The study by Daurova et al. included 22 APL patients treated with bilateral subcutaneous oligosiloxan-based Elastosil MI fillers [13]. Patients were followed up for 5 years, and researchers reported permanent results in reconstructing the defects with the elastosil MI. Another relatively recent study by Afra et al. used hyaluronic acid fillers for the lipoatrophy seen in the midface region of a patient [16]. The outcomes were maintained at the patient’s last follow-up 6 months following the procedure.

3. Flaps

Studies in which lipoatrophy was treated with flaps were case reports, excluding the study by Schmitz et al. which was retrospectively designed [11]. Flap donor sites varied, with two studies employing temporal muscle flap [14, 17], another for a radial forearm flap [18], two studies utilizing the transverse rectus abdominis myocutaneous (TRAM) flap [19, 20], two free anterolateral thigh flaps [21], two paddles of TAP adiposal flaps [22]. Two studies reported follow-up corrective operations [18, 23]. All studies but one has reported satisfactory results [11]. In the study by Schmitz et al. 4 patients were treated with free flap surgery, and although cutaneous flaps were reported to demonstrate better results than myocutaneous flaps, the study concluded that relative patient results were worse compared to patients undergoing a lipofilling procedure due to the ptosis and atrophy seen following the free flap operations [11]. The study by Guelinckx et al., researchers used two free anterolateral thigh flaps and reported markedly improved facial appearance [21]. Notably, microscopic examination of the facial arteries and veins revealed vasculitis and perivasculitis bilaterally and signs of inflammation in both patients undergoing the operation.

4. Lipofilling and Autologous Fat Transfer

Another highly preferred method for APL was lipofilling, assessed by two retrospective studies and other case reports included in this review [11, 12]. Despite the fat resorption seen in the follow-ups, patients reported satisfaction and acceptance towards repeated treatment sessions. Autologous fat transfers are of importance for APL patients, especially as they have increased fat deposition in the lower body, which makes it an easy donor site for most patients who wish to resolve the facial and breast tissue atrophy, as well as loose excess fat tissue in the thighs [8, 23, 24].

5. Other Procedures

Rhytidectomy was the operation of choice in the study by Davies et al. Two patients, aged 47 and 63, presented to the clinic with facial lipodystrophy and excess fat accumulation in the trochanteric region [25]. Both patients underwent rhytidectomy procedures, which were relatively difficult due to the lack of facial subcutaneous fat. One of those patients received a dermis graft to the nasolabial folds and oral commissures. Satisfactory results were reported for both patients.

## Discussion

In the current review, we aimed to present the different operations utilized to treat the facial lipoatrophy seen in APL patients, and how we treated three patients at our institution. Facial lipoatrophy as it is seen in APL is more common among young female patients and the early onset of the disease impacts the affected child or adolescent’s self-image negatively [7, 8, 11, 15]. It should not be dismissed as this could lead to a plethora of mental issues in the developing youth [11]. In all the studies included, before treatment, patients had reported distress regarding their appearance and how it affected their personal and social lives, with some sharing how their employment status was also adversely affected [7, 8].

The loss of fat tissue in the face is commonly associated with cachexia, which is especially the typical manifestation in AIDS patients [26]. This similarity automatically allows for a false attribution of various characteristics to BSS patients, which are related to the stereotypes imposed on the HIV (+) population. Thus, the disease not only poses individual psychosocial problems and self-image disturbances but also serious socio-economic consequences on afflicted patients’ lives due to prejudices they face. Hence, following the operations we reviewed, most patients reported satisfaction and improved life quality, which links back to improved social status. With this regard, the authors uphold the necessity of treatment for BSS patients, highlighting the suffering caused by the decreased quality of life (QoL). Accessibility of treatment plays one of the most potent roles in alleviating this issue, as treatments for BSS are not covered by insurance and are considered solely cosmetic. The literature and the cases we presented prove this argument untrue. A parallel of this scenario was argued for in the near history: breast reconstruction following breast cancer. We observed a shift in how operations for reconstructing defects caused by breast cancer and its treatments are perceived. Currently, breast reconstruction surgeries for breast cancer patients are included in insurance, as consensus on the necessity of such procedures was established on grounds of patients’ increased life quality and better mental status following a devastating disease.

The young age with which APL presents may help with its recognition by physicians, as it is unusual for facial lipoatrophy to occur at that age, and necessary work to differentiate it from other potential underlying causes is vital [27]. Despite the trochanteric pattern of fat accumulation commonly described in BSS, patients presenting with facial lipodystrophy reported how their close circle mistaken the underlying cause of their appearance as anorexia nervosa, which is far more commonly encountered in young women [8, 20]. The same patient related how this misattribution had reached the magnitude of unnecessary admissions to hospitals [8]. The role of the physician here is highlighted in the vitality of accurate diagnosis and prevention of sequelae of faulty treatments and the economic burden of misdirected healthcare sources. Thus, diagnosis of BSS requires a detailed investigation of the patient history of past infections and the presence of other autoimmune diseases [4, 28]. Of importance as a marker is hypocomplementemia (low serum C3), which aids the physical examination in establishing a diagnosis [5, 27]. With all that is yet to be understood with regard to the pathogenesis and systemic effects of this disease, the renal damage and other metabolic disorders that accompany this variant of lipodystrophy require quick action of detection, assessment, and treatment [27].

When approaching treatment of choice for BSS patients, it is important to consider individual variations among patients. Patients included in this review—as well as patients presenting to our institution—were of different ages (pediatric population to upper middle age) and phenotypes (underweight, overweight, the anatomical border the lipoatrophy reached). The latter has serious implications on the donor site and type of flap/graft to be chosen, or whether extraneous materials would be the appropriate choice [27]. Opting for autologous fat grafts is the preferred approach for most patients. However, in a setting in which fat grafts cannot be obtained—such as extremely low fat reserves, pediatric populations, or other contraindications—alloplastic materials prevail as a superior choice as compared to bone—cartilage grafting. In our study, this was the case for patient 1 (a 7-year-old) and patient 2 (who presented with extreme lipoatrophy and no available fat deposits). Accordingly, we selected Medpor and spongiose bone grafts, respectively. The latter method is not among the primarily preferred interventions due to the challenges associated with bone harvesting, as well as the increased morbidity and mortality linked to this procedure [29].

For all treatments, it is crucial that they were performed after the lipoatrophic process has come to a halt. Allowing for the transferred tissue to atrophy would require the repetition of tedious operations, which presents a set of challenges for the patients and surgeons alike.

The success of the treatment studied was measured based on the preserved transferred tissue/injected fillers in the follow-ups. In almost all treatments, a degree of tissue resorption is considered normal due to physiologic processes. The studies with the longest duration of follow-up were by Daurova et al. and Goosesens et al. , five and eleven years respectively [13, 20]. Both studies reported the success and permanency of the procedures they used, which were TRAM flap and the elastosil MI. subcutaneous fillers. Studies in which lipofilling (autologous fat grafts) was the treatment of choice demonstrated marked resorption requiring multiple sessions to maintain the results [8, 12, 24], yet patients showed acceptance as those two procedures are less invasive compared to the free-grafting and flap surgeries, which required general anesthesia and extensive postoperative care [11].

Most studies included in this review opted for flaps to correct facial lipoatrophy [11, 14, 18–20, 22, 23]. The utilization of flaps demanded surgical expertise as anastomosis of the delicate facial arteries and veins with the flap bed required precision. The study by Guelinckx et al. reported vasculitis and perivasculitis of the facial arteries and veins bilaterally for both of the patients who underwent anterolateral thigh flaps operation [23]. One of the patients had an arterial thrombosis which required repair of the anastomosis below the mandibular margin, after which the inflammation of the vasculature was microscopically detected. Despite these complications, the desired results were achieved, and patients were satisfied with the appearance.

When it comes to flaps as a choice for resolving the lipoatrophic areas in the face, it is paramount to choose the right donor site. Donor site morbidity, scarring, the degree of asymmetry following the implantation of the flap—depending on the bulkiness of the tissue—and potential follow-up trimming surgeries are all factors to carefully run through prior to the procedure. Three out of eight studies opting for flaps required follow-up procedures such as liposuction or trimming due to over-correction in the first operation or gravitational migration of the flap [18, 22, 23]. However, the main advantage of appropriate flap treatments is the longevity of the results, mainly because unlike when fat is utilized as the main tissue replacement, resorption is not a regularly encountered issue, and proper techniques for flap placements can prevent atrophy of the transferred tissue [17, 19, 20]. It is important to consider the symmetricity of the results, as mild asymmetry is not uncommon. The study by Endo et al. underlined the importance of using one flap to achieve symmetrical results [18]. Follow-up procedures to fix the asymmetry, whether operational or non-operational, are an option [11].

Another highly preferred method for correcting the defects was lipofilling. This method yielded pleasing results according to patients. Although fat resorption occurs with time requiring follow-up treatment sessions, patients’ satisfaction rates were high, especially considering that donor sites were regions of excess fat accumulation, as is common with BSS. Patients were more likely to undergo the treatment again when compared with flap operations according to the study by Schmitz et al. [11]

An advantage proposed in the same study was that the uniformity of distribution and non-ptosis when contrasted with the flap procedures provided relatively better results. For autologous fat grafts, it is standard to observe 40–60% resorption of the grafted fat depending on the region and individual susceptibilities in the patients. As such, resorption rates are minimized when the fat is injected into its natural location, as it is in the buccal area and breast tissue. The true remaining grafted fat can be seen around 6 months following the procedure, as the surgeon should account for the edema and additional material included along with the fat grafts, such as plasma and other cellular components.

Dermal Fat Auto-grafts (DFA) were not highly nor recently preferred. Ower et al. and Hurwitz et al. relied solely on DFA from the abdomen and buttocks, Endo et al. employed DFA to support the radial forearm flap. Patient satisfaction was reported in all three studies, though a follow-up trimming procedure was required in two studies [7, 18]. The follow-up durations ranged between 20–36 months, with prevailing patient satisfaction despite the mild asymmetry noted. It is important to note that the utilization of dermal fat grafts was gradually replaced by lipofilling throughout the past two decades in the field, as the unpredictable resorption rates of these grafts and the technical difficulties of the procedure along with the complications—reactions due to the glandular structure, increased risk of infections and further scarring, the incidence of epithelial cysts—rendered it less favorable [30]. Indeed, the three studies using dermal fat grafts were from a minimum of three decades ago.

Little insight was provided with regards to fillers, with only two studies, one opting for Elastosil MI. and a more recent one opting for hyaluronic acid. The main advantage of employing fillers is the minimal invasiveness of the procedure when compared to other techniques included in this study [16]. This factor makes it more feasible and accessible for patients who are hesitant to undergo operative treatment, as well as patients of a younger age, as we presented in one of our patients (Fig. 1A, B). Further studies to investigate the permanency and immunogenicity of the exogenous filling materials are needed to reach a comprehensive conclusion, especially considering the pathophysiology in these patients and the increased incidence of accompanying autoimmune disorders. Though biodegradable HA fillers are FDA-approved for midface defects, the longevity of these fillers in BSS patients across different patient populations needs further research [16]. No further data regarding Elastosil MI was retrievable in the literature (Table 3).

**Table 3:** Advantages and disadvantages for the treatments in the included studies

Study	Treatment	Advantage	Disadvantage
Ower et al. [7]	Autologous free dermal fat graft from the abdomen	Abundance of donor site.	The delicacy of the graft poses increased risk of losing it.
Heidmann et al. [8]	Autologous fat grafting harvested from the lower extremities and injected into the face and breast.	Readily available, inexpensive, enables a natural feeling and does not cause adverse immunological reactions.	The durability of autologous tissue grafts is uncertain in patients with BSS due to the progressive nature of the lipoatrophy, and multiple treatments may be necessary to obtain sustained acceptable results.
Schmitz et al. [11]	Free flap surgery	–	The anastomoses remained difficult and were a real challenge. The difficulty of these anastomoses was not only the incongruence of the vessel diameter but also the presence of crooked and inflamed facial vessels (vasculitis and perivasculitis). All free flaps developed ptosis or atrophy and needed some further treatment to guarantee a fairly lasting result.
	Lipofilling	Patients expressed willingness to undergo the procedure again. Atrophy, following lipofilling was uniform and ptosis did not develop, yielding better results as compared to free flap surgeries.	The results are unpredictable, and the injected fatty tissue has variable viability.
Cortese et al. [12]	Cheek and zygomatic bridge augmentation through autologous free fat transplantation (lipofilling)	The maxillofacial region is suitable for lipofilling due to its rich vascularity.	The results are unpredictable, and the injected fatty tissue has variable viability.
Daurova et al. [13]	Bilateral subcutaneous injection Elastosil MI.	Permanency of results observed over five years.	No further data available.
Serra et al. [14]	Temporal muscle flap	Filling of the bilateral defects symmetrically	No further data available.
Hurwitz et al. [15]	Autologous free dermal fat graft from the buttocks and a following trimming operation	Simultaneous bilateral approach to minimize asymmetry and scars in the donor and recipient sites.	The graft demonstrated ptosis on the left side. Central fat plugs were prominent and unnaturally firm. Required corrective trimming procedure.
Afra et al. [16]	Hyaluronic acid filler	Hyaluronic acid fillers contain biodegradable, cross‐linked HA gel with efficacy and safety. HA filler injection is a non‐operative volumization and sculpting technique.	-
Van der Wal et al. [17]	Temporal muscle flap	–	The dissection is relatively easy, minimal scarring. good symmetry. The scars are invisible compared to those produced by local flaps, tubular de-epithelialized flaps and free vascularized dermis-fat flaps. The temporalis muscles do not show atrophy in contrast to fat grafts, dermis-fat grafts, and fascia-fat and dermis-fascia-fat grafts.
Endo et al. [18]	Double paddle dermis-fat radial forearm free flap, and for the small areas that required augmentation but were out of reach of the flap, free dermal fat grafts were used.	Reconstruction of both sides of the face in a short period of surgical time. The radial forearm flap is thin, so the postoperative facial bulk is lighter compared with flaps from the lower body. Vascular pedicle condition is safe and reliable.	Six months later the patient returned for the second stage operation because some gravitational migration of the flap had occurred and trimming of the drooping parts was necessary. The donor site defect on the forearm was covered with a split-thickness skin graft but resulted in a little hypertrophic scarring. The volume of subcutaneous tissue obtained from the forearm is limited. Thus, in the case of a patient with severe lipodystrophy, the volume of tissue available for reconstruction with this flap might be inadequate to correct the deformity.
Coessens et al. [19]	Transverse rectus abdominis myocutaneous flap.	The muscle consistency is maintained in the long-term. The volume of the rectus muscle is ideal to fill the cheek area, and the de-epithelialized portion of the flap helps to create a smooth contour. flaps can be harvested simultaneously, leaving a low abdominal scar. The sizable vascular pedicle makes the anastomoses with facial vessels expeditious. Simultaneous correction of both sides with stable facial appearance over time and acceptable donor site morbidity are among its main advantages.	The fullness of the lower face is not in perfect harmony with the bizygomatic contour, probably because of a lack of suspension of the flaps to the malar area.
Goossens et al. [20]	Two transverse rectus abdominis myocutaneous flaps.	Eleven years following reconstruction, the result is satisfactory and the muscle volume is unchanged.	A flap failure on one side would ruin the whole reconstruction as adequate symmetry would not be established after a salvage procedure with another type of flap. An important disadvantage of scapular/parascapular flaps is the necessity of changing the position of the patient during the operation. The vascular pedicle is short, and the dissection is time-consuming and tedious.
Guelinckx et al. [21]	Two free anterolateral thigh flaps. For the second patient, six months after the transfer additional liposuction at the mandibular border was done to improve the cheek line.	The fat tissue of this flap is not affected by the disease and is redundantly present. The flap is easy to remodel and shows little tendency toward ptosis. Moreover, additional sculpturing by means of liposuction is possible in later stages. Finally, pre-elevation of the flap simultaneously allows for symmetrical contour restoration of the fat hypertrophy at the lower extremities that is frequently present with this disease.	Possible redundancy of the fat tissue required following liposuction, which could be prevent by extensive preoperative thinning of the flap. Inflammation of the facial vessels may be seen.
Okazaki et al. [22]	One—stage bilateral restoration of the facial contour with one donor site and a single pair of vascular anastomoses (Two paddles of TAP adiposal flaps based on hemilateral thoracodorsal vessels).	The bilateral facial atrophy can be augmented simultaneously with only one donor site. It requires only one pair of vascular anastomoses, reducing the surgical time. Most of the latissimus dorsi muscle and horizontal branch of thoracodorsal nerve can be spared. The harvest of TAP flap along the descending branch does not leave a prominent scar if viewed from either the front or the back because it lies near the midaxillary line.	–
Jeon et al. [24]	Autologous fat transfer into the face and breast.	Autologous fat transfer is a safe and minimally invasive method that yields good aesthetic outcomes without functional compromise, while maintaining natural facial expressions and contour and has become the established as a mode of facial soft tissue restoration. By harvesting from the lower limbs in these patients, it serves to remove the excess fat deposition that can occur in conjunction with BSS.	The patient experienced some fat resorption in the cheek area after the last procedure. The main limitation to this procedure is fat resorption, which remains unpredictable and inevitable.
Davies et al. [25]	For the first patient, facial rhytidectomy, upper lip chemical peel, upper and lower blepharoplasties. For the second patient, facial rhytidectomy and dermis graft, septorhinoplasty and blepharoplasty.	-	The second patient's grafting procedure was difficult due to the lack of subdermal fat.

*BSS* Barraquer Simons Syndrome, *HA* Hyaluronic Acid, *TAP* Thoracodorsal Artery Perforator

Rhytidectomy and other rejuvenation operations such as blepharoplasties were the operations of choice in the study by Davies et al., as the patients had a milder form of lipoatrophy and were older adults, with sagging accompanying the lack of subcutaneous fat [25]. The results were satisfactory, but it is important for plastic surgeons considering these operations in older BSS patients to realize the complexity of operating with minimal to no underlying fat tissue, which demands enhanced expertise as compared with operating on normal skin.

## Conclusion

The current review explored, analyzed, and presented various operative treatment modalities for facial reconstruction in patients with BSS. The marked lipoatrophy seen in the facial area, especially accompanying the patient profile, mainly in female adolescents to middle-aged women directly impacts their quality of life through societal, interpersonal, and personal means. The variety of treatments was weighed with their respective advantages and disadvantages. It is important to choose the appropriate procedure for the patient based on patient characteristics, even more, to guide them through the treatment of choice based on their end goals.
